# Brain morphology correlates of learning and cognitive flexibility in a fish species (*Poecilia reticulata*)

**DOI:** 10.1098/rspb.2022.0844

**Published:** 2022-07-13

**Authors:** Zegni Triki, Maria Granell-Ruiz, Stephanie Fong, Mirjam Amcoff, Niclas Kolm

**Affiliations:** Department of Zoology, Stockholm University, Svante Arrheniusväg 18 B, Stockholm, Sweden

**Keywords:** cognition, cognitive flexibility, learning, telencephalon, optic tectum, cerebellum

## Abstract

Determining how variation in brain morphology affects cognitive abilities is important to understand inter-individual variation in cognition and, ultimately, cognitive evolution. Yet, despite many decades of research in this area, there is surprisingly little experimental data available from assays that quantify cognitive abilities and brain morphology in the same individuals. Here, we tested female guppies (*Poecilia reticulata*) in two tasks, colour discrimination and reversal learning, to evaluate their learning abilities and cognitive flexibility. We then estimated the size of five brain regions (telencephalon, optic tectum, hypothalamus, cerebellum and dorsal medulla), in addition to relative brain size. We found that optic tectum relative size, in relation to the rest of the brain, correlated positively with discrimination learning performance, while relative telencephalon size correlated positively with reversal learning performance. The other brain measures were not associated with performance in either task. By evaluating how fast learning occurs and how fast an animal adjusts its learning rules to changing conditions, we find support for that different brain regions have distinct functional correlations at the individual level. Importantly, telencephalon size emerges as an important neural correlate of higher executive functions such as cognitive flexibility. This is rare evidence supporting the theory that more neural tissue in key brain regions confers cognitive benefits.

## 1. Introduction

Variation in brain morphology is ubiquitous at all taxonomic levels [1]. This variation often correlates with various aspects of cognitive and behavioural performance. For instance, positive associations have been found between improved cognitive/behavioural abilities and overall brain size [1–10], brain region sizes [11–16], neuron cell numbers [17] and potentially neural connectivity [18]. Most of the evidence on the brain–cognition relationship have emerged from the comparative phylogenetic approach [1,12,19]. For instance, MacLean *et al*. [7] found a positive correlation between absolute brain size and cognitive performance in self-control tasks across various vertebrate species. Moreover, by studying closely related species of gobies with distinct space use, White & Brown [20] found that rock-pool gobies were more successful than sand-dwelling gobies in finding their way home after translocation. This advantage was later found to be associated with performance in a spatial learning task [21], and with larger telencephalon size [22]. While we know much about the braincognition relationship at the species level, little is known about this relationship at the individual level. Hence, additional studies that focus on more detailed aspects of individual brain morphology and cognitive performance are valuable to increase our understanding of whether phenotypic neural plasticity also yields cognitive benefits, much like evolutionary changes at the species level.

Fish are a suitable study taxon to address this issue, both for their brain anatomy, which is compartmentalized into main regions with distinct functions [23] and for their often surprisingly high cognitive abilities [24–26]. In terms of the specific functions of the major brain regions in teleost fish, the olfactory bulbs receive olfactory sensory input and relay it to the telencephalon; the telencephalon is considered the main centre for cognition and decision making; the optic tectum receives visual sensory input and relays it to the telencephalon; the hypothalamus regulates many basic functions but also motivation and some aspects of social behaviour; the cerebellum controls mainly motor coordination abilities but also aspects of cognition; and the dorsal medulla controls autonomic functions [23,27–32]. Still, quantitative evidence of to what extent the relative tissue volume of different brain regions may affect individual cognitive abilities remains largely unknown. Furthermore, studies within species have rarely looked at the link between the different brain region sizes and cognitive performance in the same individuals (e.g. [15,33]). Therefore, we investigate the relationship between brain region size and cognitive abilities in the same individuals, focusing on individual cognitive performance in a reversal learning test.

The reversal learning test is a commonly used paradigm to assess learning abilities and cognitive flexibility across species and taxa [10,34–37]. Here, we used a standard reversal learning test design in small fish consisting of two parts [e.g. 10]. The first part is a two-colour discrimination learning task that tests whether the individual can associate a colour cue with a food reward and how fast it can reach a learning criterion. Once the individual has learnt the cue-reward association, the reward contingency is reversed, and the previously unrewarded colour becomes the new rewarded cue. The first colour-discrimination learning task thus tests for associative learning ability, while the reversal learning task tests the animal's ability to adapt and change its behaviour after the reversal of the cue-reward, a measure of behavioural and cognitive flexibility [35,38,39]. This capacity is one of the three core executive functions in vertebrate cognition, where the other two are inhibitory control and working memory [38]. In addition, executive functions are often defined as control mechanisms of general purpose that modulate different cognitive subprocesses and are thus highly ecologically relevant [40].

Using this reversal learning paradigm, we tested individual performance in discrimination learning and reversal learning in female guppies, *Poecilia reticulata*. These fish were from the third generation of an ongoing artificial selection experiment, where fish were selected for having a larger or smaller telencephalon in relation to the rest of the brain [41] (see 'Material and methods'; electronic supplementary material). Upon accomplishing the tasks, we measured the brain morphology of all the individually tested fish and estimated total brain size and the size of five major brain regions: telencephalon, optic tectum, hypothalamus, cerebellum and dorsal medulla. We thus aimed to explore the correlation between brain region volumes and individual performance in the two cognitive tasks. Since the telencephalon is known for its involvement in various perceptual and cognitive functions, like spatial cognition [22], inhibitory control abilities [16], memory and decision-making [32,42–44], we expected to find a positive relationship between telencephalon size and individual performance in the two tasks. Given that much less is known about the involvement of the other quantified brain regions in cognition, we avoided making any predictions about the presence or direction of these regions' effects on individual performance in discrimination learning or cognitive flexibility.

## 2. Material and methods

### (a) Study animals

We conducted this study between February and April 2020 at the Stockholm University Zoology department fish laboratory facilities. We tested 66 female guppies belonging to replicated laboratory selection lines of Trinidadian guppies (*Poecilia reticulata*). These lines had been artificially selected for relatively larger and smaller telencephalon sizes for three generations (see electronic supplementary material). We housed the 66 female guppies (33 up-selected and 33 down-selected) individually in experimental aquaria (length×width×height; 40 × 15 × 15 cm) enriched with 2 cm gravel and artificial plant and continuously aerated water. The experimental aquaria had two guillotine doors, one transparent and one opaque, dividing each aquarium into two compartments: the housing and the test compartment [see 10]. The laboratory and the experimental room had an ambient temperature of approximately 26°C with a light schedule of 12 h light and 12 h dark. We fed the guppies *ad libitum* with fish flakes and newly hatched brine shrimp six days per week. During the learning tasks, fish acquired food solely from test trials. Unfortunately, seven out of the 66 fish were found dead on the floor after jumping out of the experimental tanks during the night. To avoid potential experimenter bias, a person not involved in this study concealed the true identity of the tested fish (selection line) with running numbers throughout the experiment.

### (b) Cognitive tasks

#### (i) Training

The protocol we used here for the colour discrimination learning tasks followed Buechel *et al*. [10]. The test paradigm consisted of a two-choice task where fish had to learn to associate a food reward with a colour cue (i.e. yellow versus red). For this, we placed a white tablet with 20 small wells (10 mm diameter and 5 mm depth) in every experimental aquarium at the bottom of the test compartment. Only two wells (always the same wells) were used repeatedly for food rewards throughout the experiment. On top of these two wells, we placed two small plastic discs (14 mm diameter), one red and one yellow. Underneath each disc, we put one defrosted adult artemia. Only one food item was accessible to the fish if the latter dislodged the disc by pushing it sideways to uncover the well. The other food item was inaccessible by covering it with a disc having a small silicone knob preventing it from being dislodged sideways. Before any colour cue discrimination learning could start, the fish needed to learn how to dislodge a disc sideways to uncover the food reward. For this, we performed 12 training trials over two consecutive days. We used a black disc during the pre-training to cover the food item only partially. We gradually reduced the gap over trials until the disc covered the well entirely, and all individual fish successfully retrieved the food item by dislodging the disc sideways.

#### (ii) Colour discrimination task

After all the fish had learnt to retrieve food underneath a removable disc, we tested their abilities in the colour discrimination learning task. To control for (and later test) potential colour and/or side biases, we trained half the fish to associate the yellow disc with the food reward, while the other half were trained to associate the red disc with the food reward, and we randomly presented the rewarded cue on the left or right side in each trial. Fish received three trials (i.e. one session) per day over two weeks, with no tests on the weekends. In every test trial, we scored a choice as ‘correct’ if a fish chose the rewarded colour in its first attempt, and we scored a choice as ‘failure’ if a fish chose the wrong colour in its first attempt. If a fish chose the wrong disc in the first attempt, we allowed them to go to the rewarded disc and retrieve the food item from the correct disc to ensure positive reinforcement in each trial [see 10]. To evaluate individual fish performance, we set two conservative alternative learning criteria. To fulfil them, an individual fish had to score either ten correct choices out of twelve consecutive trials (i.e. during four sessions of three trials each) or six correct choices out of six consecutive trials (i.e. during two sessions of three trials each). With a binomial test, these criteria meant that the probability of learning the task by chance was *p* < 0.05.

#### (iii) Reversal learning task

Out of *n* = 59 tested fish, we had *n* = 58 that successfully learned the discrimination learning task within 30 trials. We tested these fish in a reversal-learning task where the reward contingency from the discrimination task was reversed. For example, fish that successfully learnt to associate the yellow disc with a food reward in the discrimination learning task now had to unlearn that association and learn to associate the red disc with a food reward (and vice versa for the other colour combination) in the reversal learning task. In total, we ran 66 trials of reversal learning for each individual over 4.5 weeks (with no tests on the weekends). Performance evaluation was made according to the same criteria as in the discrimination learning task. It is unlikely that repeated fish training over learning trials affected their brain plasticity since a previous study found no such short-term effects in the guppy [45].

### (c) Brain morphology

As mentioned previously, seven fish died during the experiment by jumping out of the aquarium overnight. We prepared the remaining 59 female guppies for brain measures by first euthanizing them with an overdose of benzocaine (0.4 g 1^−1^) and then fixating their whole bodies in 4% paraformaldehyde phosphate-buffered saline (PBS) for 5 days. Upon fixation, we washed the samples twice in PBS for 10 min each before storing them at 4°C pending brain dissections. With a digital calliper, we measured fish standard length (SL) to the nearest 0.01 millimetre (*n* = 59, mean ± s.d.: 28.11 ± 1.12 mm).

For brain morphology measures, we first dissected the whole brain out of the skull, and photographed the brain from the dorsal, right lateral, left lateral and ventral view by employing a stereo zoom microscope Leica MZFLIII with a digital camera Leica DFC 490. Second, we estimated the length (*L*), width (*W*) and height (*H*) of the telencephalon, optic tectum, cerebellum, dorsal medulla, hypothalamus and olfactory bulb with the open-access software ImageJ. Third, we fitted the *L*, *W* and *H* measures in an ellipsoid function (1) (based on [46], and [47]), and calculated the volume (*V*) of every brain region (in mm^3^):

2.1 $$\;\begin{array}{r} V=(L\;\times \;W\;\times \;H)\;\frac{\pi }{6}. \end{array}\;$$

### (d) Data analysis

We used the open-access software R v. 3.6.3 [48] to run all statistical analyses and generate the figures. Given that the selection lines had only been under directional selection for telencephalon size for three generations, they had not yet reached a level of significant difference in females' relative telencephalon size between the lines at the time we performed this study (in males, this effect was evident already after three generations [16], and at later generations, this effect became evident in both sexes [41]). Therefore, after confirming at the group level that there were no significant differences in telencephalon size or cognitive performance between the up-and down-selected lines (see electronic supplementary material for further details), we focused instead on the individual level performance and its neural correlates.

As predictor variables in our analyses, we had six brain measures: relative brain size and the relative sizes of the telencephalon, optic tectum, hypothalamus, cerebellum and dorsal medulla. Specifically, to account for potential effects of body size, we fitted brain size (volume in mm^3^) as a continuous predictor and body size (SL in mm) as a control covariate (log-transformed and standardized with the *scale* function [49]). Similarly, to establish the relative size of the included five brain regions, we fitted the size of the brain region of interest as a continuous predictor and the size of the rest of the brain without that brain region as a control covariate (all volumes in mm^3^ were log-transformed and standardized). To test learning performance, we used survival analyses with the Cox proportional hazards models (*coxph* function from R package *survival*). This type of model perfectly fits the current study's aims, where ‘death’ in the classic survival analyses can be replaced by ‘success’ in learning tasks. Success and failure and time to succeed in the discrimination learning task and the reversal learning task were thus fitted in *Coxph* models. In addition, every *Coxph* model had selection line as a categorical predictor to control for potential group effect. Also, to account for potential colour bias toward the discs employed in the two learning tasks (red and yellow) and the effect of selection line replicate, we added the factor ‘colour’ and ‘replicate’ as a cluster to the *Coxph* models. We used the functions *ggeffect* and *ggpredict*, from R package *ggeffects*, to plot model predictions from *Coxph* models. For survival analysis models, the *ggpredict* plots depict ‘risk score’ or risk of ‘death’ on the y-axis, which should be read as the occurrence of ‘success’ in the learning tasks. Finally, we checked that all *Coxph* models met the proportional hazards assumptions using the *cox.zph*. For further details, please check our step-by-step code provided along with the data via the shared link in the 'Data accessibility' section below.

## 3. Results

**Figure 1.**
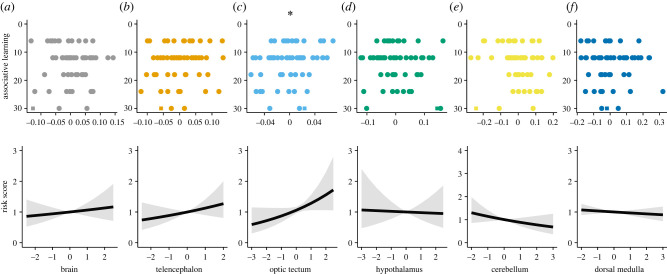
Individual brain morphology and performance in the discrimination learning task. Scatterplots of the number of trials needed to reach the learning criterion in the discrimination learning task by the six brain measures. Upper panels show inverted *y*-axes to facilitate visual assessment of performance where fewer trials to reach criterion means faster learning abilities. Also, on the *x*-axes of the upper panels, for visual simplicity, we used residuals from the regression relationship of log-brain size on log-body size for brain measure, and residuals from the regression relationship of log-brain region size on log-size of the rest of the brain for brain region measures. The lower panels show Cox proportional hazards model predictions of the relationship between success score in the discrimination learning task and the *z*-standardized six brain measures. A higher ‘Risk score’ indicates a higher success rate. The grey area indicates the 95% CI. Circle datapoints are individuals who successfully learned the discrimination learning task within a maximum of 30 trials, while the one square datapoint refers to the only fish that failed to learn. *Coxph: n* = 59 female guppies; *p < 0.05. (Online version in colour.)

**Figure 2.**
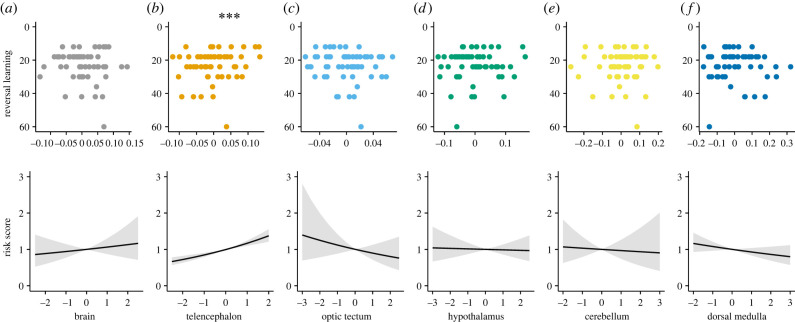
Individual brain morphology and performance in the reversal learning task. Scatterplots of the number of trials needed to reach the learning criterion in the reversal learning task by the six brain measures. Upper panels show inverted *y*-axes to facilitate visual assessment of performance where fewer trials to reach criterion means faster learning abilities. Also, on the *x*-axes of the upper panels, for visual simplicity, we used residuals from the regression relationship of log-brain size on log-body size for brain measure, and residuals from the regression relationship of log-brain region size on log-size of the rest of the brain for brain region measures. The lower panels show Cox proportional hazards model predictions of the relationship between success score in the reversal learning task and the *z*-standardized six brain measures. A higher ‘Risk score’ indicates a higher success rate. The grey area indicates the 95% CI. Circle datapoints are individuals who successfully learned the reversal task within a maximum of 66 trials. *Coxph: n* *=* 58 female guppies; ***p *<* 0.001. (Online version in colour.)

Our findings show that among the brain measures investigated here, relative optic tectum size correlated positively with individual discrimination learning abilities (*n* = 59, hazard ratio (HR) = 1.124, *p* = 0.038, 95% CI [1.01, 1.51]; figure 1). In the reversal learning, on the other hand, relative telencephalon size emerged to be the best explanatory variable of individual learning speed and success rate (*n* = 58, HR = 1.174, *p* < 0.001, 95% CI [1.10, 1.25]; figure 2). The results further revealed that larger relative telencephalon size correlated with faster learning and proportionally more success in the reversal learning task (figure 2). Neither relative brain size nor the size of the other brain regions significantly explained performance in the discrimination and reversal learning tasks (all *p* ≥ 0.2; table 1).

**Table 1.** Summary table for the statistical outcomes of the six models. Statistically significant outcomes with *p-*value*s* ≤ 0.05 (alpha set at 0.05) are indicated in bold type. HR: hazard ratio. 95% CI: lower and upper bounds of the 95% confidence interval.

brain measure	discrimination learning				reversal learning			
	*n*	HR	95% CI	*p*-value	*n*	HR	95% CI	*p*-value
total brain	59	1.063	0.88, 1.30	0.544	58	0.897	0.72, 1.10	0.311
telencephalon	59	1.13	0.89, 1.42	0.300	58	**1.174**	**1.10, 1.25**	**<0.001**
optic tectum	59	**1.124**	**1.01, 1.51**	**0.038**	58	0.955	0.71, 1.13	0.349
hypothalamus	59	0.979	0.74, 1.28	0.878	58	0.986	0.85, 1.14	0.856
cerebellum	59	0.879	0.71, 1.08	0.219	58	0.967	0.74, 1.26	0.806
dorsal medulla	59	0.968	0.89, 1.05	0.461	58	0.927	0.83, 1.04	0.188

Although we did not detect any significant statistical outliers in the data, as shown in figure 2, one data point (fish ID no. 57 with reversal learning performance = 60) can potentially be viewed as an outlier. We therefore performed further analyses without this data point and excluding it did not affect the conclusions (electronic supplementary material, figure S3).

## 4. Discussion

We asked which neural substrates best explain the cognitive processes of colour discrimination learning ability and cognitive flexibility. The optic tectum and telencephalon emerged as key regions positively associated with individual discrimination and reversal learning abilities, respectively. But the other brain measures did not predict individual performance.

The first outcome of our study is the effect of optic tectum size on colour discrimination learning ability. The size of this brain region has, to our knowledge, not previously been identified as a potential cause of variation in discrimination learning, but the salience of a cue strongly influences how effectively it will be associated with a reward during discrimination learning [50,51]). Hence, based on the general importance of the optic tectum in visual processing [52], we suggest that the positive association between optic tectum size and discrimination learning observed here is due to the role of the optic tectum in determining the perceived salience of a cue [33,43,52,53]. However, once the reward contingency was reversed in the reversal task, the optic tectum seemingly did not have an important role in an individual's flexibility to adjust to the new conditions. Broadly defined, cognition includes all ways by which animals take in information through the senses, process, retain and act on it [54]. Our findings here support that the optic tectum facilitated colour discrimination learning through increased perception of the visual cues, while the absence of its effect on cognitive flexibility suggests that visual information alone was not enough to reverse the choice. Instead, the individual needed more complex information processing to update its decision-rules and replace the previous colour-reward association with a new one. Indeed, the positive association between telencephalon size and performance in the reversal learning task supports that view.

Our findings are in line with what has been discovered in lesion experiments. Such studies have demonstrated how fish with the entire telencephalon removed may keep several aspects of their behavioural repertoire and solve simple classical conditioning [55], while they fail to solve more complex tasks like reversal learning [56]. This agrees with the general view that behavioural flexibility, a part of the general intelligence tool kit [38], is typically located in the prefrontal cortex in mammals [see review by 39] and the neostriatum in birds [57], homologs of the fish telencephalon [32]. To our knowledge, our findings are among the first to document an association between individual telencephalon size and cognitive flexibility within a species. However, the exact anatomical changes that underlie the variation in telencephalon size in the sampled fish are yet to be revealed. Hence, the differences in telencephalon size might be due to either the number of neurons [58], changes in connectivity [15] or both. Either way, the observed variation in telencephalon size yielded correlated cognitive differences in our experiments.

We did not find any link between the size of the hypothalamus, dorsal medulla or cerebellum and cognitive performance. This supports that the hypothalamus is probably more involved in autonomic functions and social cognition and not cognition *per se* [15,59,60]. For the cerebellum, the absence of predictive power in the two learning tasks suggests that these tasks do not require much motor control or emotional learning (see reviews in [29,61]; but see [62]). Similarly, we did not find any relationship between the dorsal medulla and cognitive performance. Also, this makes sense given the known role of this region in autonomic functions [27].

Relative brain size did not predict learning performance in our experiments. Although selection experiments on brain size have shown that larger brains often yield higher cognitive performance, including in reversal learning [2,10,63], we were unable to find similar patterns in our data. Artificial selection experiments on total brain size create large variation in brain size between up- and down-selected lines, making it possible to detect group-level differences in performance. Such differences may be challenging to detect in laboratory-reared populations without directional selection on brain size or cognitive ability directly linked to brain size. Captive-kept animals in low complexity environments and under relaxed selection for cognitive abilities associated with predation and foraging often have dramatic reductions in brain size [64–67]. We speculate that individual brain regions are less sensitive to such domestication effects, because even though there may be trade-offs between investment into different brain regions [30], the energetic costs of individual regions are lower than for the entire brain [8,41]. Because of this, correlations between brain region size and cognitive ability might be possible to reveal also in captive kept populations (see also [68] for further discussion on this).

This study tested females from the third generation of up- and down-selected fish for relative telencephalon size. Using males from the third generation telencephalon selection lines, with significant differences in telencephalon size between up- and down-selected fish, Triki *et al*. [16] recently showed that up-selected fish with larger telencephalons outperformed down-selected fish with smaller telencephalons in a self-control task (inhibitory control task). Moreover, after four generations of selection both sexes displayed substantial significant differences in relative telencephalon size between the large- and the small-telencephalon selected lines [41]. In the present analysis of females from the third generation of selection, the group-level analyses showed no difference in telencephalon size or cognitive performance (electronic supplementary material). Nevertheless, the individual-level analyses revealed positive correlations between cognitive performance and the relative size of the telencephalon and the optic tectum, potentially driven by individual variation from the two lines (see discussion by Fong *et al*. on how artificial selection experiments directed such individual variation in relative telencephalon size [41]). Thus, the current results on individual-level telencephalon size and performance nicely complement those previous group-level findings in male guppies showing cognitive benefits from a larger telencephalon.

To conclude, we find that out of the six brain measures investigated here (relative brain size, relative size of telencephalon, optic tectum, hypothalamus, cerebellum and dorsal medulla), optic tectum and telencephalon size were most strongly correlated to colour discrimination learning and reversal learning at the individual level, respectively. Our study highlights the importance of variation in brain region size and its role in underpinning individual variation in cognitive abilities. We propose that simultaneously studying individual brain morphology and cognitive ability (as well as other aspects of behavioural variation) can be an important approach for increasing our understanding of the mechanisms behind variation in animal behaviour in general, and cognition in particular.

## Data Availability

The data and the R code to reproduce the statistical outcomes and generate the figures are available in the Figshare repository (https://doi.org/10.6084/m9.figshare.14406320). Electronic supplementary material is available online [70].
